# *In Vitro* Antifungal Activity of Sanguinarine and Chelerythrine Derivatives against Phytopathogenic Fungi

**DOI:** 10.3390/molecules171113026

**Published:** 2012-11-02

**Authors:** Xin-Juan Yang, Fang Miao, Yao Yao, Fang-Jun Cao, Rui Yang, Yan-Ni Ma, Bao-Fu Qin, Le Zhou

**Affiliations:** 1 College of Science, Northwest A&F University, Yangling 712100, Shaanxi, China; Email: yxjsn2@163.com (X.-J.Y.); mumu2006yo@126.com (Y.Y.); caofangjun@yahoo.com.cn (F.-J.C.); yrer@126.com (R.Y.); ni-2003@163.com (Y.-N.M.); 2 College of Life Science, Northwest A&F University, Yangling 712100, Shaanxi, China; Email: miaofangmf@163.com (F.M.); baofu_qin@yahoo.com.cn (B.-F.Q.)

**Keywords:** sanguinarine, chelerythrine, quaternary benzo[*c*]phenanthridine alkaloids, antifungal activity, phytopathogenic fungi

## Abstract

In order to understand the antifungal activity of some derivatives of sanguinarine (**S**) and chelerythrine (**C**) and their structure-activity relationships, sixteen derivatives of **S** and **C** were prepared and evaluated for *in vitro* antifungal activity against seven phytopathogenic fungi by the mycelial growth rate method. The results showed that **S**, **C** and their 6-alkoxy dihydro derivatives **S_1_**–**S_4_**, **C_1_**–**C_4_** and 6-cyanodihydro derivatives **S_5_**, **C_5_** showed significant antifungal activity at 100 µg/mL against all the tested fungi. For most tested fungi, the median effective concentrations of **S**, **S_1_**, **C** and **C_1_** were in a range of 14–50 µg/mL. The structure-activity relationship showed that the C=N^+^ moiety was the determinant for the antifungal activity of **S** and **C**. **S_1_**–**S_5_** and **C_1_**–**C_5_** could be considered as the precursors of **S** and **C**, respectively. Thus, the present results strongly suggested that **S** and **C** or their derivatives **S_1_**–**S_5_** and **C_1_**–**C_5_** should be considered as good lead compounds or model molecules to develop new anti-phytopathogenic fungal agents.

## 1. Introduction

The continuing development of fungicidal resistance in plant and human pathogens necessitates the discovery and development of new fungicides. In the past decades, natural product-based plant protectants have attracted a lot of attention from researchers owing to the fact they are perceived to have lower environmental and mammalian toxicity [1].

**Figure 1:**
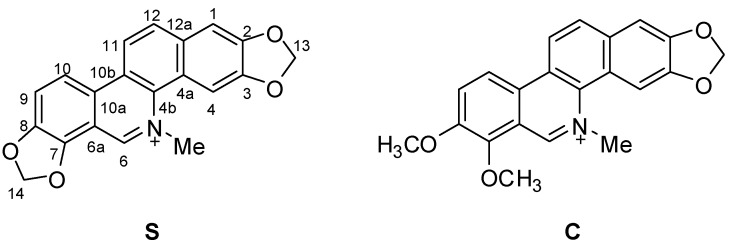
Structures of sanguinarine (**S**) and chelerythrine (**C**).

Natural quaternary benzo[*c*]phenanthridine alkaloids (QBAs) constuitute a relative small class of isoquinoline alkaloids that nevertheless are widely distributed in the higher plant families Fumariaceae, Papaveraceae and Rutaceae [2,3]. Among QBAs, sanguinarine (**S**) and chelerythrine (**C**) (Figure 1) are the most common and their richest natural sources are the plants *Sanguinaria Canadensis* L., *Dicranostigma lacucoides* Hook.f. & T. Thoms., *Chelidonium majus* L., *Macleaya*, *Bocconia* species from the Papaveraceae family and some members of *Zanthoxylum* (Rutaceae). In the past decades, QBAs had attracted much attention from investigators because of their extensive and important bioactivities, which include antitumour [4,5], antimicrobial [6,7,8,9], anti-inflammatory [10], antiviral [11], anti-HIV [12], antiparasitic action against *Trichodina* sp. [13], *Dactylogyrus intermedius* [14] and malaria [15], anti-platelet aggregation [16], anti-angiogenesis [17] and anti-acetylcholinesterase properties [18]. We recently also found that 1-alkoxydihydro derivatives of **S** had significant acaricidal activity against *Psoroptes cuniculi* [19].

Previous research had proven that **S** and **C** had significant activities against plant microbial pathogens. As early as in the period of 1939 to 1973, S had been found to be fungistatic on several plant fungal pathogens including *Phytomatotrichum omnivorum*, *Sclerotium rolfisii*, *Gaeumannomyces grminis*, *Rhizoctonia solani*, *Amillaria mellea*, *Fusarium oxysyporum* and *Verticillium alboatrum* [20,21,22]. In 1999, Matos *et al*. demonstrated that **S** and **C** were the active antifungal components of the plant extracts from *Chelidonium majus* L. or *Macleaye cordata* (Willd) R. Br. against 13 strains of phytopanthogenic fungi from the genus *Fusarium* [23]. The results were further confirmed by Liu *et al*. using bioassay-guided fractionation of the extract of *Macleaya cordata* R. Br. [24]. In addition, the extract from *Macleaye cordata* (Willd) R. Br. was able to effectively control powdery mildew (*Sphaerotheca pannosa* var. *rosae*) of greenhouse roses *in vivo* [25]. However, until now the activity of the derivatives of **S** and **C** against phytopathogenic fungi and their structure-activity relationships were not reported. The objective of the present study was to systematically evaluate the anti-phytopanthogenic fungal activity of a series of the derivatives of **S** and **C** and understand their structure-activity relationships.

## 2. Results and Discussion

### 2.1. Chemistry

**Figure 2:**
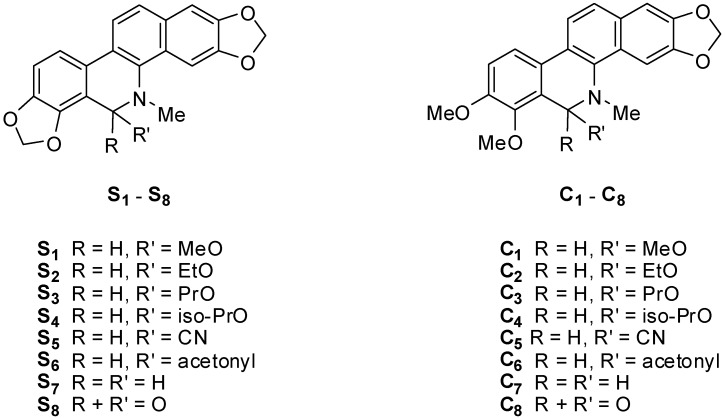
Derivatives of sanguinarine **S_1_–S_8_** and chelerythrine **C_1_–C_8_**.

**Scheme 1:**
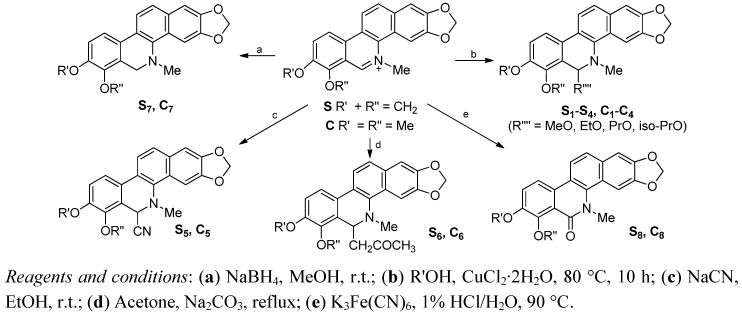
Synthetic pathway for compounds **S_1_–S_8_** and **C_1_–C_8_**.

Compounds **S** and **C** were obtained by isolation from the entire plant of *Macleaya microcarpa* (Maxim) Fedde according to the method reported by us [26] and used as the starting materials to synthesize **S_1_–S_8_** and **C_1_–C_8_** (Figure 2), respectively, using the synthetic route outlined in Scheme 1.

Structural modifications of **S** and **C** included introduction of alkoxyl, cyano and acetonyl at C-6 by nucleophilic addition, and the reduction and oxidation of the C=N double bond at the C-6 position. Compounds **S_1_–S_5_/C_1_–C_5_** and **S_6_–S_8_/C_6_–C_8_** were prepared according to the methods recently reported by us [19,26].

The structures of all compounds were elucidated by spectroscopic analyses including ESI-MS, ^1^H- and ^13^C-NMR spectra. The spectral data of all the compounds were in agreement with that previously reported by us [19,26]. It should be noted that **S_1_–S_6_** and **C_1_–C_6_** were racemates and used as such for the bioactivity assays. We tried to resolve the racemates to their enantiomers but were unsuccessful. The main reason is that these compounds very easily convert back to their corresponding parent compounds **S** or **C** under acidic or even weakly acidic conditions.

### 2.2. Antifungal Activity

#### 2.2.1. Screening of Antifungal Activity *in Vitro*

The *in vitro* antifungal activities of **S**, **C** and their derivatives at a concentration of 100 μg/mL were assayed by the linear growth rate method. Thiabendazole (TBZ), a commercial fungicide, was used a control. The results are shown in Table 1.

**Table 1:** Linear Growth inhibitory rates (means ± S.D.%) of 18 compounds against seven phytopathogenic fungi (100 µg/mL).

Compd.	Seven Tested Phytopathogenic Fungi *						
	*C.L.*	*V.M.*	*F.S.*	*F.O.N.*	*F.O.V.*	*P. O.*	*A.A.*
**S**	**78.6 ± 3.4**DE **	**84.8 ± 0.8**BC	**81.4 ± 1.0**BC	**72.1 ± 3.1**E	**82.0 ± 2.2**D	**60.4 ± 2.1**D	**71.2 ± 0.0**C
**S_1_**	**80.8 ± 2.0**BCDE	**88.1 ± 0.4**AB	**83.8 ± 2.6**BC	**86.6 ± 1.5**C	**84.0 ± 0.0**CD	**70.8 ± 2.1**C	**74.6 ± 0.9**C
**S_2_**	**87.5 ± 2.8**A	**85.8 ± 5.9**BC	**85.4 ± 2.6**B	**69.7 ± 1.7**E	**85.6 ± 1.6**CD	**70.1 ± 3.2**C	**70.0 ± 2.3**C
**S_3_**	**86.1 ± 0.8**AB	**93.2 ± 0.6**A	**86.9 ± 4.6**AB	**87.6 ± 2.3**C	**87.2 ± 1.6**C	**69.4 ± 1.2**C	**73.6 ± 0.9**C
**S_4_**	**78.1 ± 1.5**DE	**84.6 ± 1.8**BC	**82.0 ± 2.1**BC	**79.6 ± 5.4**D	**84.5 ± 1.0**CD	**61.1 ± 1.2**D	**64.9 ± 1.5**D
**S_5_**	**79.7 ± 0.8**CDE	**80.1 ± 0.4**C	**73.3 ± 0.0**D	**63.2 ± 0.0**F	**71.6 ± 0.7**E	**47.0 ± 0.6**E	**44.9 ± 1.6**E
**S_6_**	19.1 ± 2.0HI	−4.0 ± 3.5FG	−0.1 ± 3.2F	−0.5 ± 0.9K	−4.4 ± 0.9J	9.7 ± 1.2HI	16.2 ± 1.5H
**S_7_**	33.0 ± 1.3F	−8.3 ± 1.7H	7.5 ± 1.9E	2.0 ± 3.4JK	−0.1 ± 1.8HI	20.8 ± 0.0G	21.2 ± 0.9G
**S_8_**	18.0 ± 1.3I	3.4 ± 0.7DE	6.3 ± 0.7E	0.5 ± 0.9JK	2.6 ± 3.9H	5.8 ± 1.2IJ	11.0 ± 1.5I
**C**	**81.5 ± 2.2**BCDE	**88.5 ± 2.0**AB	**87.3 ± 0.2**AB	**85.6 ± 0.9**C	**94.2 ± 0.0**B	**74.3 ± 1.2**BC	**83.7 ± 1.8**B
**C_1_**	**79.9 ± 3.5**CDE	**93.0 ± 1.7**A	**92.4 ± 0.4**A	**94.0 ± 0.0**B	**100.0 ± 0.0**A	**77.1 ± 2.1**AB	**85.8 ± 0.9**AB
**C_2_**	**76.3 ± 0.8**E	**86.9 ± 0.4**AB	**83.4 ± 0.2**BC	**100.0 ± 0.0**A	**95.7 ± 3.7**AB	**79.9 ± 1.2**AB	**86.8 ± 0.9**AB
**C_3_**	**85.7 ± 5.1**ABC	**89.8 ± 2.1**AB	**86.4 ± 6.4**AB	**94.0 ± 0.0**B	**100.0 ± 0.0**A	**81.9 ± 2.4**A	**87.3 ± 0.9**AB
**C_4_**	**82.6 ± 4.0**ABCD	**83.5 ± 0.8**BC	**82.2 ± 0.9**BC	**90.5 ± 0.9**BC	**92.0 ± 0.0**B	**83.0 ± 0.6**A	**88.8 ± 0.9**A
**C_5_**	**80.8 ± 0.3**BCDE	**84.4 ± 0.4**BC	**78.7 ± 0.4**CD	**40.2 ± 3.4**G	**69.1 ± 1.1**E	**48.4 ± 1.1**E	**62.5 ± 0.8**D
**C_6_**	5.7 ± 3.4J	−1.4 ± 2.8EF	8.6 ± 3.9E	5.0 ± 0.9IJ	−3.3 ± 3.3IJ	1.4 ± 2.4J	5.5 ± 1.5J
**C_7_**	18.0 ± 1.3I	5.1 ± 6.9D	10.5 ± 3.2E	13.4 ± 0.0H	12.7 ± 2.4F	28.9 ± 5.9F	47.9 ± 5.9E
**C_8_**	24.0 ± 2.0GH	−5.2 ± 2.4FG	10.5 ± 1.3E	9.5 ± 4.8HI	6.8 ± 0.9G	11.9 ± 1.3H	24.7 ± 1.8G
TBZ ***	27.1 ± 1.5G	88.4 ± 0.8AB	83.9 ± 3.5BC	100.0 ± 0.0A	100.0 ± 0.0A	15.3 ± 6.7GH	30.6 ± 4.0F

* *C.L.*: *Curvularia lunata*; *V.M.*: *Valsa mali*; *F.S.*: *Fusarium solani*; *F.O.V.*: *Fusarium oxysporum f.* sp. *vasinfectum*; *A.A.*: Alternaria alternate; *P.O.*: Pyricularia oryza; *F.O.N.*: *Fusarium oxysporum* sp. niveum. ** The differences between data with different capital letters within a column are significant for the same tested fungus (*p* < 0.01) with respect to **S** and its pseudoalcoholates or **C** and its pseudoalcoholates. *** TBZ: thiabendazole.

The results in Table 1 show that among all the derivatives only the 6-alkoxydihydro derivatives **S_1_–S_4_, C_1_–C_4_** and 6-cyanodihydro derivatives **S_5_, C_5_** displayed significant activities (40.2%–100% inhibitory rate) against all seven tested fungi at 100 µg/mL. On the contrary, the other derivatives **S_6_–S_8_, C_6_–C_8_** gave lower or no activity at the same concentration. With respect to *C. lunata*, *P. oryzae* and *A. alternate*, **S, C** and all of their pseudoalcoholates (compounds **S_1_–S_4_, C_1_–C_4_**) were much more active than TBZ, a commercial fungicide (*p* < 0.01). For *V. mali* and *F. solani*, **S, S_1_–S_4_, C** and **C_1_–C_4_** showed the same activities as TBZ (*p* > 0.01). For *F. oxysporum* sp*. niveum*, *F. oxysporum* f. sp*. vasinfectum*, *P. oryza*, and *A. alternate*, the activities of **C** and its pseudoalcoholates **C_1_–C_4_** were significantly stronger than that of **S** and its corresponding pseudoalcoholates **S_1_–S_4_** (*p* < 0.01). However, for the other three fungi, no significant differences between **S** or **S_1_–S_4_** and **C** or **C_1_–C_4_** was observed (*p* > 0.01). In addition, in most cases, there was also no significant difference between the activities of pseudoalcoholates **S_1_–S_4_** or **C_1_–C_4_** and their corresponding parent compound **S** or **C** and between the different pseudoalcoholates **S_1_–S_4_** or **C_1_–C_4_** from the same parent compound **S** or **C** (*p* < 0.01).

#### 2.2.2. Antifungal Toxicity

Based on the results above, **S_1_** and **C_1_** were used as representative pseudoalcoholates to further determine their regression equations and median effective concentration (EC_50_ values) towards the seven tested fungi. **S** and **C** as parent compounds were used as control. The results are listed in Table 2. The inhibition rates of the four compounds increased as the concentration increased. All the compounds showed a significant linear correlation between the inhibition rate and log[concentration] value (*R*^2^ values = 0.9193–0.9925, *p* < 0.01). **C** and **C_1_** gave the highest activity against *C. lunata* with EC_50_ values of 15.43 µg/mL (32.5 µM) and 14.23 µg/mL (37.5 µM), respectively, and **S** and **S_1_** showed the lowest activity against *P. oryza* with EC_50_ values of 101.6 µg/mL (2291.2 µM) and 96.63 µg/mL (265.9 µM). The other EC_50_ values were in a range of 24.28 to 53.8 µg/mL. Comparison of the EC_50_ values of the four compounds for the same fungus showed that **C** and **C_1_** were more active than **S** and **S_1_** for *C. lunata*, *F. solani*, *F. oxysporum* sp*. niveum*, *P. oryzae* and *A. alternate.* However, for *F. oxysporum* f. sp*. vasinfectum* and *V. mali*, **S** and **S_1_** were more active than **C **and **C_1_**. These results were not exactly the same as those obtained from Table 1. This is because the various compounds had different slope values. On the other hand, compared the EC_50_ value (µM) of **S** (or **C**) with that of **S_1_** (or **C_1_**) for the same fungi, it was found that in most cases **S** (or **C**) was slightly more active than its correspongding pseudoalcoholate **S_1_** (or **C_1_**)*.*

Besides EC_50_ value, the slope value (*k*) in a toxicity regression equation is also an important factor for evaluation of bioactivity of a compound. A slope value reflects the concentration effect (CE) of a compound on its bioactivity. The *k* values in Table 2 showed that the CEs of **S, S_1_, C** and **C_1_** on the activities against the seven fungi were different. In order to comprehensively compare the activities of the different compounds, the value of *k*/EC_50_ (nM) of each the compound, here named as comprehensive activity (CA), was calculated and shown in Table 2. Comparing the CA values of the compounds for each fungus, it may be obviously seen that for most of the fungi both **C** and **C_1_** were more active than **S** or **S_1_**. However, the CAs of **S** or **C** were close to that of its pseudoalcoholate **S_1_** or **C_1_**. This conclusion is basically consistent with that obtained from Table 1.

**Table 2:** Toxicity regression equations and EC_50_ values of compounds **S, C, S_1_** and **C_1_** against seven fungi.

Fungus	Compd.	Toxicity regression equation *	*R*^2^	EC_50_ value		CI 95% ** (µg/mL)	CA ***
				(µg/mL)	(µM)		
C.L.	S	*y* = 0.5758*x* − 0.3905	0.9628	35.20	76.6	35.13–35.27	16.4
	S_1_	*y* = 0.5452*x* − 0.2960	0.9848	28.84	79.4	28.81–28.85	18.9
	C	*y* = 0.3413*x* + 0.0944	0.9621	15.43	32.5	15.21–15.65	22.1
	C_1_	*y* = 0.3635*x* + 0.0808	0.9914	14.23	37.5	14.18–14.28	25.5
V.M.	S	*y* = 0.6317*x* − 0.3751	0.9901	24.28	52.9	24.24–24.32	26.0
	S_1_	*y* = 0.5275*x* − 0.2329	0.9860	24.51	67.5	23.97–25.05	21.5
	C	*y* = 0.6918*x* − 0.6038	0.9505	39.40	82.9	39.30–39.50	17.6
	C_1_	*y* = 0.6420*x* − 0.4423	0.9904	29.36	77.4	29.34–29.38	21.9
F.S.	S	*y* = 0.7415*x* − 0.6922	0.9717	40.53	88.3	40.46–40.60	18.3
	S_1_	*y* = 0.5949*x* − 0.4189	0.957	35.04	96.4	34.95–35.13	17.0
	C	*y* = 0.5005*x* − 0.2123	0.9600	26.50	55.8	26.41–26.59	18.9
	C_1_	*y* = 0.5949*x* − 0.3540	0.9925	27.26	71.8	27.24–27.28	21.8
F.O.N.	S	*y* = 0.5139*x* − 0.3676	0.9768	48.79	106.2	48.73–48.85	10.9
	S_1_	*y* = 0.5487*x* − 0.3317	0.9705	32.79	90.2	32.73–32.85	16.7
	C	*y* = 0.6186*x* − 0.4076	0.9891	29.32	61.7	29.30–29.34	21.1
	C_1_	*y* = 0.6629*x* − 0.4624	0.9836	28.30	74.6	28.26–28.34	23.4
F.O.V.	S	*y* = 0.6007*x* − 0.4137	0.9735	33.20	72.3	33.14–33.26	18.1
	S_1_	*y* = 0.6320*x* − 0.4735	0.9826	34.70	95.5	34.66–34.74	18.2
	C	*y* = 1.0309*x* − 1.2677	0.9600	51.84	109.1	51.80–51.88	19.9
	C_1_	*y* = 0.6303*x* − 0.4308	0.9684	29.98	79.0	29.91–30.06	21.0
P.O.	S	*y* = 0.3182*x* − 0.1386	0.9687	101.6	221.2	101.2–102.00	3.1
	S_1_	*y* = 0.4238*x* − 0.3413	0.9914	96.63	265.9	96.61-96.63	4.4
	C	*y* = 0.7447*x* − 0.7550	0.9727	48.44	101.9	48.41–48.47	15.4
	C_1_	*y* = 0.7178*x* − 0.6779	0.9843	43.75	115.3	43.72–43.78	16.4
A.A.	S	*y* = 0.7921*x* − 0.8663	0.9608	53.08	115.6	53.03–53.13	14.9
	S_1_	*y* = 0.5176*x* − 0.3453	0.9193	43.47	119.6	43.10–43.84	11.9
	C	*y* = 0.9270*x* − 1.0756	0.9572	50.08	105.4	50.03–50.13	18.5
	C_1_	*y* = 0.6218*x* − 0.4104	0.9659	29.11	76.7	29.04–29.18	21.4

* *y*: Inhibitory rate. *x*: Log_10_[concentration(mg/L)]; ** CI 95%: Confidence interval at 95% probability (µg/mL). *** Slope value/EC_50_ (μg/mL) × 1,000.

### 2.3. Structure-Activity Relationship

Unlike the parent compounds **S** or **C**, all the derivatives of **S** or **C** lack the C=N^+^ moiety, and their only structural difference lies in their different substituents at the 6 position. **S_7_** and **C_7_** were the reduction products of **S** and **C**, respectively. The significant difference of the activities between **S** and **S_7_** or **C** and **C_7_** showed that the iminium moiety in the molecule **S** or **C** is the determinant for their antifungal activity.

**S_1_–S_4_** or **C_1_–C_4_** have *N*,*O*-acetal structures and both **S_5_** and **C_5_** are structurally α-hydroxynitrile derivatives. All these compounds are able to easily convert back to the corresponding parent compound **S** or **C** under acidic conditions [27,28], including the slightly acidic environment of the lysosome of cells [29]. Unlike **S_1_–S_5_** and **C_1_–C_5_**, compounds **S_6_–S_8_** and **C_6_–C_8_** could not undergo a similar transformation under the same conditions. The significant difference in activities between **S_1_–S_5_** and **S_6_–S_8_** or between **C_1_–C_5_** and **C_6_–C_8_** as well as the lack of a significant difference between the of **S_1_–S_5_** or **C_1_–C_5_** strongly suggest that the activities of **S_1_–S_5_** and **C_1_–C_5_** might well come from their corresponding hydrolytic products, *i.e.*, **S** or **C**. In other words, **S** and **C** might be the real active compounds of **S_1_–S_5_** and **C_1_–C_5_**, respectively, while **S_1_–S_5_** and **C_1_–C_5_** might be only the precursors of **S** and **C**. In addition, comparing the activities of **C** with that of **S** or **C_1_–C_5_** with **S_1_–S_5_**, we could find that for most of the fungi, a 7,8-methoxy group, relative to a 7,8-methylenedioxy group, could enhance the activity to a certain degree. It was worth noting that the structure and antifungal activity relationships were very similar to the case of their reported antibacterial activities [26].

## 3. Experimental

### 3.1. General

Sanguinarine iodide (**S**) and chelerythrine iodide (**C**) were obtained in our laboratory by isolation from the whole plant of *M. microcarpa* (Maxim) Fedde [24]. Melting points (m.p.) were determined on XT-4 micro-melting point apparatus and are uncorrected. ^1^H-NMR and ^13^C-NMR spectra were recorded with a Bruker AVANCE Ⅲ instrument operating at 500 and 125 MHz, respectively, using TMS as an internal standard. ESI-MS was measured on a Trace mass spectrometer. Thiabendazole (TBZ, ≥99.1%), a commercial fungicide standard, was purchased from Sigma-Aldrich Trading Co. Ltd. (Shanghai, China). Dimethyl sulfoxide (DMSO) was purchased from J&K China Chemical Ltd. (Beijing, China). Other reagents were obtained locally and of analytical grade. The water used was redistilled and ion-free. *Curvularia lunata*, *Alternaria alternate*, *Fusarium solani*, *Fusarium oxysporum* sp. *vasinfectum*, *Valsa mali*, *Fusarium oxysporum* sp. *niveum* and *Pyricularia oryzae* were isolated, identified and provided by the Center of Pesticide Research, Northwest A&F University, (Shaanxi, China). These fungi were grown on PDA plates at 28 °C and maintained by periodic subculturing at 4 °C.

### 3.2. Synthesis of ***S_1_–S_8_*** and ***C_1_–C_8_***

Compounds **S_1_–S_5_** and **C_1_–C_5_** were prepared according to the methods and the procedure recently reported by us [19]. Compounds **S_6_–S_8_** and **C_6_–C_8_** were prepared the procedure according to the methods previously reported by us [26]. The spectral data of **S_1_–S_8_** and **C_1_–C_8_** are consistent with our previous papers [19,26].

### 3.3. Screening of Antifungal Activity *in Vitro*

The antifungal activity *in vitro* was assayed by the growth rate method [30] with slight modifications. The tested fungi maintained on potato-dextrose-agar (PDA) medium slants were subcultured for 48 h in Petri dishes prior to testing and used for inoculation of fungal strains on PDA plates. The tested compounds were completely dissolved in DMSO, and then diluted by water to provide the stock solution (1.0 µg/mL) in 5% DMSO aqueous solution. The stock solution was completely mixed with the autoclaved PDA medium to provide a medium containing 100 µg/mL of sample and then poured into the Petri dishes in a laminar flow chamber. Thiabendazole solution (1.0 µg/mL) in 5% DMSO aqueous solution and 5% DMSO aqueous solution were used as the treated control and the untreated control, respectively. When the medium in the plates was partially solidified, a 5-mm thick and 4-mm diameter disc of fungus cut from earlier subcultured Petri dishes was placed at the centre of the semi-solid medium. The dishes were kept in an incubator at 28 °C for 72 h. Each experiment was carried out in triplicates. The diameters (in mm) of inhibition zones were measured in three different directions and the growth inhibition rates were calculated according to the following formula and expressed as means ± S.D.:

Growth inhibition rate (%) = [(*d*_c_ − *d*_0_) − (*d*_s_ − *d*_0_)]/(*d*_c_ − *d*_0_) × 100

where *d*_0_: Diameter of the fungus cut, *d*_c_: Diameter of the untreated control fungus, *d*_s_: Diameter of the sample-treated fungus.

### 3.4. Toxicity Assays

Based on *in vitro* antifungal activity screening results, **S, S_1_, C** and **C_1_** were selected to determine their toxicity. According to the method described above, a stock solution containing 800 μg/mL samples was prepared in 5% DMSO aqueous solution. Then the stock solution was completely mixed with the autoclaved PDA medium to prepare a set of media containing 120, 90, 80, 40, 20, 10, 5 and 2.5 μg/mL samples, respectively. 5% DMSO aqueous solution was used as the untreated control. According to the method described above, the antifungal activity for each concentration was determined. Each experiment was carried out in triplicate. The average inhibition rate for each test was calculated. The concentration of the compound was transformed to the corresponding logarithm value (log_10_*C*). A linear regression, namely toxicity regression equation, was established by using the least square method. EC_50_ values were calculated from the toxicity regression equation.

### 3.5. Statistic Analysis

SPSS statistics V17.0 software was used to analyze the data and establish toxicity regression equationa. Duncan multiple comparison test was performed on the data to determine significant differences between the inhibition rates of different compounds at the same concentration.

## 4. Conclusions

In this study, sixteen derivatives of **S** and **C** were obtained by modification of their C=N^+^ groups and evaluated for *in vitro* antifungal activity against seven phytopathogenic fungi by the mycelial growth rate method. The structure-activity relationships were also discussed. **S, C, S_1_–S_5_** and **C_1_–C_5_** showed significant antifungal activities against all the tested fungi at 100 μg/mL. For most tested fungi, the median effective concentrations of **S, S_1_, C** and **C_1_** were in a range of 14–50 μg/mL. The iminium bond was proven to be the determinant for the antifungal activity of **S** and **C**. **S_1_–S_5_** and **C_1_–C_5_** were considered as the precursors of **S** and **C**, respectively. Based on the present results, **S** and **C** should be considered as good lead compounds or model molecules to develop new anti-phytopathogenic fungal agents.
